# Healthcare workers’ perspectives on a prescription phone program to meet the health equity needs of patients in the emergency department: a qualitative study

**DOI:** 10.1007/s43678-024-00735-y

**Published:** 2024-07-01

**Authors:** Kathryn Hodwitz, Galo F. Ginocchio, Tali Fedorovsky, Hannah Girdler, Brielle Bossin, Clara Juando-Prats, Evelyn Dell, Andrea Somers, Jennifer Hulme

**Affiliations:** 1 Applied Health Research Centre, Li Ka Shing Knowledge Institute, St. Michael’s Hospital, Unity Health Toronto, Toronto, ON Canada; 2 Einstein Lab, Department of Psychology, University of Toronto, Toronto, ON Canada; 3 Temerty Faculty of Medicine, University of Toronto, Toronto, ON Canada; 4 Centre for Global Equity in Emergency Medicine, University Health Network, Toronto, ON Canada; 5 Emergency Department, St. Michael’s Hospital, Toronto, ON Canada; 6 Dalla Lana School of Public Health, University of Toronto, Toronto, ON Canada; 7 Michael’s Hospital, University of Toronto, Toronto, ON Canada; 8 University Health Network, Toronto, ON Canada; 9 Division of Emergency Medicine, University of Toronto, Toronto, ON Canada; 10 University Health Network, Toronto General Hospital, Toronto, ON Canada; 11 Department of Family and Community Medicine, University of Toronto, Toronto, ON Canada

**Keywords:** Emergency medicine, Health equity, Digital health inequities, Disadvantaged populations, Discharge planning, Qualitative research, Program evaluation, Médecine d’urgence, Équité en santé, Inégalités en santé numérique, Populations défavorisées, Planification des congés, Recherche qualitative, Évaluation du programme

## Abstract

**Objectives:**

People experiencing homelessness and marginalization face considerable barriers to accessing healthcare services. Increased reliance on technology within healthcare has exacerbated these inequities. We evaluated a hospital-based prescription phone program aimed to reduce digital health inequities and improve access to services among marginalized patients in Emergency Departments. We examined the perceived outcomes of the program and the contextual barriers and facilitators affecting outcomes.

**Methods:**

We conducted a constructivist qualitative program evaluation at two urban, academic hospitals in Toronto, Ontario. We interviewed 12 healthcare workers about their perspectives on program implementation and outcomes and analyzed the interview data using reflexive thematic analysis.

**Results:**

Our analyses generated five interrelated program outcomes: building trust with patients, facilitating independence in healthcare, bridging sectors of care, enabling equitable care for marginalized populations, and mitigating moral distress among healthcare workers. Participants expressed that phone provision is critical for adequately serving patients who face barriers to accessing health and social services, and for supporting healthcare workers who often lack resources to adequately serve these patients. We identified key contextual enablers and challenges that may influence program outcomes and future implementation efforts.

**Conclusions:**

Our findings suggest that providing phones to marginalized patient populations may address digital and social health inequities; however, building trusting relationships with patients, understanding the unique needs of these populations, and operating within a biopsychosocial model of health are key to program success.

## Abstrait

**Objectifs:**

Les personnes sans abri et marginalisées font face à des obstacles considérables pour accéder aux services de santé. Le recours accru à la technologie dans les soins de santé a exacerbé ces inégalités. Nous avons évalué un programme de téléphones d’ordonnance en milieu hospitalier visant à réduire les inégalités en santé numérique et à améliorer l’accès aux services chez les patients marginalisés des services d’urgence. Nous avons examiné les résultats perçus du programme et les obstacles contextuels et facilitateurs qui influent sur les résultats.

**Méthodes:**

Nous avons mené une évaluation qualitative constructiviste de programmes dans deux hôpitaux universitaires urbains de Toronto, en Ontario. Nous avons interviewé 12 travailleurs de la santé au sujet de leurs points de vue sur la mise en œuvre et les résultats du programme et analysé les données des entrevues au moyen d’une analyse thématique réflexive.

**Résultats:**

Nos analyses ont généré cinq résultats de programme interdépendants : établir la confiance avec les patients, faciliter l’indépendance dans les soins de santé, rapprocher les secteurs de soins, permettre des soins équitables pour les populations marginalisées et atténuer la détresse morale chez les travailleurs de la santé. Les participants ont indiqué que la fourniture de services téléphoniques est essentielle pour servir adéquatement les patients qui font face à des obstacles à l’accès aux services de santé et aux services sociaux, et pour soutenir les travailleurs de la santé qui manquent souvent de ressources pour servir adéquatement ces patients. Nous avons cerné les principaux catalyseurs contextuels et les défis qui pourraient influer sur les résultats du programme et les efforts de mise en œuvre futurs.

**Conclusion:**

Nos résultats suggèrent que la fourniture de téléphones aux populations de patients marginalisés peut remédier aux inégalités numériques et sociales en matière de santé; cependant, établir des relations de confiance avec les patients, comprendre les besoins uniques de ces populations, La réussite du programme repose sur le fait de fonctionner dans un modèle biopsychosocial de la santé.

## Clinician’s capsule

**Table:** 

***What is known about the topic?***
Patients experiencing marginalizing conditions often lack access to phones, which can hinder connections to follow-up care and services post-discharge.
***What did this study ask?***
What are the potential outcomes associated with prescribing mobile phones to patients experiencing marginalization in the ED?
***What did this study find?***
Phone provision can build trust with patients, facilitate referrals and follow-up care, and mitigate moral distress among healthcare workers.
***Why does this study matter to clinicians?***
Prescription mobile phones may improve care post-discharge, but key contextual and operational factors must be considered for future implementation.

## Introduction

People experiencing homelessness and marginalization face considerable health inequities, including barriers to accessing healthcare and increased morbidity and mortality [1–3]. They tend to receive a disproportionate amount of care via Emergency Departments (EDs) [4–6] and have higher rates of readmission and ED revisits [5, 7, 8], largely because they lack access to primary care [9–11], and because hospital discharge and follow-up planning fail to account for their unique needs [12, 13], creating a “revolving hospital door” [14]. Increased reliance on technology in healthcare has exacerbated disparities, particularly since the COVID-19 pandemic [15–17]. This “digital divide” has resulted in disadvantaged populations facing heightened barriers to healthcare and poorer health outcomes [18]. Follow-up communication has long been challenging for people without fixed addresses or phones [19], and recent shifts to virtual care have inadvertently excluded those without access to technology or the Internet [3, 16, 20]. Thus, the concept of digital exclusion has emerged as an important determinant of health [15, 16, 21].

In response to the growing digital health inequities and communication challenges during the COVID-19 pandemic, an ED-based prescription phone program was developed to provide mobile phones to patients experiencing social and digital exclusion [22]. The program aimed to facilitate follow-up care, improve communication, and enhance connection to services for patients post-discharge. Very few phone provision interventions have been studied: one pilot program in the UK found distributing mobile phones to ED patients experiencing homelessness enabled better connection to services[23], and one study found providing cell phones or tablets to US veterans living in supportive housing helped them engage in healthcare[24]. This is the first known hospital-based program in Canada to provide free mobile phones to patients to facilitate follow-up care and access to services. In this study, we explored healthcare workers’ experiences with program implementation and outcomes, including contextual and operational facilitators and barriers. Through this, we sought to understand whether and how mobile phone provision can reduce digital health inequities among marginalized populations, and how to optimize future implementation efforts.

## Methods

### Study design and setting

PHONE-CONNECT *(Phones for Healthier Ontarians iN EDs-COvid NEeds met by Cellular Telephone)* is a hospital-based program that provides mobile phones to patients experiencing marginalization to facilitate follow-up care post ED-discharge. It was founded in March, 2020 by Emergency physicians at the University Health Network in Toronto, Ontario and expanded to two other Toronto inner city hospitals [22]. The program aims to improve patient outcomes by enabling connections to outpatient healthcare (e.g., primary care, specialist appointments) and community services (e.g., shelters, case workers). Healthcare workers in the EDs offer phones with 6-month plans to patients who do not have personal phones who they think could benefit from one. The phones are activated before discharge and the phone numbers are added to the patients’ medical records.

This study is one component of an evaluation of the PHONE-CONNECT program. Using a constructivist approach, we examined the experiences of healthcare workers involved in the program to understand how the program operated in practice. Patient perspectives and outcomes will be explored separately. Data for this study were collected from August to December, 2022 at two urban, inner city, academic hospitals in Toronto, Ontario: University Health Network and St. Michael’s Hospital.

### Participants and sampling

As part of our broader evaluation, a survey was distributed to all healthcare workers in the hospitals’ EDs to gather their views on the program. This survey was sent via email and delivered to the EDs in hardcopy by two research coordinators not involved in program administration. Respondents indicated if they had direct experience with the program and if they consented to be contacted for an interview. We purposively selected those healthcare workers involved in the program for interviews, aiming for heterogeneity of roles where possible, including physicians, social workers, and peer support workers. We recruited participants via email and offered a gift card honorarium for participation.

### Data generation and analysis

Two authors (GG and BB), familiar with the program but not known by participants, conducted in-depth interviews with participants, in person or via Zoom, using a semi-structured guide. Interviews focused on participants’ experiences with the program, perspectives on program outcomes, views on how and why the program worked, including contextual factors influencing outcomes [25], as well as implementation challenges, strengths, and suggestions for improvement. Interviews lasted 30–60 min, and were audio recorded and transcribed verbatim.

Two other authors (KH and CJP), external to the program and experienced in qualitative research, conducted reflexive thematic analysis [26] using a narrative approach wherein the unit of analysis was stories told by participants [27]. Analysis involved first reading the transcripts fully, then coding them inductively, identifying and refining themes, and producing interpretive findings based on the themes [26]. KH first analyzed five transcripts to develop initial codes and analytical memos, which were then refined through discussions with CJP. A draft thematic framework was developed to describe perceived outcomes and contextual enablers and challenges associated with the program. This framework was iteratively updated through analysis of the remaining transcripts and discussions between analysts (KH and CJP). The full team reviewed the themes for coherence and resonance at two points during analysis to provide clinical expertise and contextual perspectives to the findings. Analysis was deemed complete when thematic sufficiency was reached.

We engaged in reflexivity throughout data generation and analysis, acknowledging how our unique lenses and roles as clinicians or healthcare researchers may have shaped the research process. Utilizing a lead analyst external to the program helped enable an openness to the data, and using large, unfragmented segments of narrative data during analysis helped mitigate inadvertently misrepresenting participants’ meanings in our interpretation.

### Ethics

We received ethics approval from the University Health Network Research Ethics Board (Protocol 20-5505) and a formal waiver from the St. Michael’s Hospital Research Ethics Board.

## Results

**Fig. 1:**
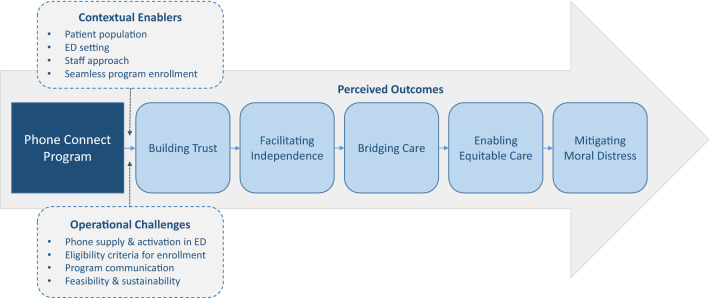
Perceived program outcomes, contextual enablers, and operational challenges

Participants were 12 healthcare workers (demographic characteristics in Table 1). We identified five main interrelated program outcomes, as well as key program enablers and challenges. These findings are described below and depicted in Fig. 1, with additional supporting quotes in Tables 2 and 3.

**Table 1:** Participant demographic characteristics

Demographic characteristic	*n*
Gender	
Male	1
Female	10
Non-binary	1
Age (range)	33–53
Years in practice (range)	3–25
Professional role	
Peer support worker	6
Social workers	4
Emergency physicians	2
Ethnicity	
White	6
South Asian	2
Preferred to not answer	4

**Table 2:** Perceived program outcomes

Theme	Data excerpts
Building trust & connection	▪ “[The phone is] a tool to [say], ‘Hey, I want to build a relationship with you… I care [and] we're going work on this. I'm serious about my commitment to helping you through your situation.’” SMH-001 ▪ “Introducing the phone program to [patients]… it really opens up dialogue, like we are actually trying to connect with you: “We’re offering you this, so you can come connect with us.”” SMH-002 ▪ “I think it’s pretty special to be able to give someone something that has value to them, is something that could be lost, or could be broken, but you're not going through like “I don’t trust you to use this valuable thing”, you're just saying “This is something I'm going to give it to you.” I think that's pretty powerful.” UHN-002 ▪ “I helped [the patient] get back into his shelter… and then gave him phone numbers of rehabilitation places… He finally got into [one], and now he’s completed his stay, he’s housed, he’s working, and he will call me once in a while just to [ask questions]… So although I’m not serving him anymore… it’s given him a place to fall back on.” SMH-002
Facilitating independence	▪ “Sometimes they’ve been homeless for so long, that they lose the confidence… to kind of do it themselves. So you show them a couple of times… and the next time they're able to do it themselves. So, it’s… building some confidence within them to help themselves.” SMH-002 ▪ “I have one [patient] who was afraid to make phone calls herself and now she is halfway through school, a certificate program. She’s gotten two jobs… So, you can see how people are broken down by poverty, homelessness, and then how something as simple as a phone and a connection to somebody who's going to advise them, how that can actually change the person's confidence and perspective.” SMH-002 ▪ “I feel like unhoused people, when they do get a phone number, it becomes more than a way to connect to them. They now have a new sense of identity. Or [sense of] just being in the game. ‘I'm somewhat closer to being more stable, and like everyone else’.” SMH-001 ▪ “If this [program] was not available, it would have been difficult [for the patient to overcome addiction]. One of the reasons why you see people going back to those things… is because of lack of communication or lack of connection.” SMH-007
Bridging care	Connecting to services: ▪ “There’s all these teams [in ERs] that are like, “Oh, here’s support”. But then [the patient says], “okay I’m being discharged back to my street or back to my shelter”, so it kind of ends at the institution… And the phone kind of bridges that… Now, they have a way to call the services that you've provided them… So it’s like a bridge for them for health care.” SMH-002 ▪ “Phone-Connect became a monumental tool… because there’s a lot of people who, you can make an appointment for them, but they can’t follow-up… allowing someone to have that opportunity where they can access the phone, they can be reached, they can make those connections, it’s pivotal, It changes a lot of things for people.” SMH-004 ▪ “It promotes connection with multiple services… So when I make a referral… if they have a phone number, then that referral is much more likely to be a success. I’m talking about referrals to housing, case management, other health services, other clinics, family physicians, services in the community… having a phone in this day and age is really critical to functioning and accessing any kind of service.” UHN-004 ▪ “We have patients who have cardio follow-ups or have specialist follow-ups, [and] the clinics typically don’t make appointments on the spot. They usually call you with your appointment date and time. And our patients, if they don’t have a phone, they'll never get that follow-up appointment… which can lead to really significant health risks in the future.” SMH-003 ▪ “I got him a SIM card that same day. He secured a shelter the next morning… he was able to reach out, update his community housing file, and now he has a housing offer, and he’s likely going to be housed in the next month.” SMH-001 Reducing ED visits and admissions: ▪ “[Patients given phones] presented [to the Emergency Department] less actually. Because the phone, [in the] majority of the cases is their main obstacle. As long as they have the phone, they can connect to resources, and they wouldn’t really need any other supports from the hospital.” SMH-005 ▪ “Once somebody comes in and connects with us, we’re able to connect them to other health services so that they don’t have to utilize the [emergency department] for their primary health care.” SMH-002 ▪ “If we can connect people to phones who need case worker support, specialist support, and GP support… accessing those will limit further presentations and potential admissions in the future.” SMH-003
Enabling equitable care	▪ “Without phones, I don't know how we can serve this population and I don’t know how we can connect to this population. Especially with the challenges we’ve been facing with COVID… I think it’s very difficult for people to survive out there without phones.” SMH-002 ▪ “Particularly for people who have experiences of trauma or homelessness, who spent a lot of time waiting in lines, who often have a lot of experience of being ignored in health care, expecting someone to be able to sort of wait patiently in a busy room for several hours is not a realistic expectation. And that's what we have to do if we can't arrange a follow-up time with someone. So being able to say like ‘Show up at this place, at this time, they're going to call you and tell you exactly what to do.’ [is] really, really helpful.” UHN-002 ▪ “We give out safety planning booklets, and of course we go over it with the patients, but so much of it says ‘have 911 one on speed dial, have your cell phone accessible, always have your cell phone charged’, and giving that booklet to somebody who doesn’t have a cell phone who’s in immediate danger, just seems really, really silly.” SMH-003
Mitigating moral distress	▪ “Oftentimes the phone has been able to act as like a tool for so many different patient situations. I can’t even express how helpful it's been in our role.” SMH-003 ▪ “[Our job] has to do with connection, it has to do with helping people. A phone program I would say is really positive. It helps make our job easier, especially when you have client… and you want to be able to offer some support. Maybe housing support, or addiction support. It's frustrating when you’re not able to reach them. Most of our NFA [No Fixed Address] populations are without phones. Most times their phones are stolen because they are living on the street, it's kind of rough for them out there. We always come across patients that don't have any means of communication… Having a phone program is really helpful to be able to connect with them, to be able to follow up.” SMH-007 ▪ “It makes our job easier. And it gives a kind of fulfillment. You're doing something that you can see. I'm happy that I’m able to support, or help someone… when they receive the phones, some of them are so excited. Some find it hard to believe… You can see how important that service is to them. So yeah, it’s a good feeling.” SMH-007 ▪ “There’s so much moral distress, and so much burnout in emergency medicine, because we see the consequences of poverty and… we can so seldom do anything about it. So for providers, when you offer people a tool… that potentially is going to have a profound impact for someone… people find it really refreshing.” UHN-002

**Table 3:** Contextual enablers and operational challenges

Theme	Data excerpts
Contextual enablers	
Patient population	▪ “[The people who enrolled in the program are] generally people who are precariously housed. Maybe they’re in a shelter, maybe they’re staying on the street… And they’re feeling disenfranchised from their community and from their family, not being able to connect with people… to help them with their day to day functioning [or]… specifically for follow up medical care and the referral that’s been made.” SMH-006 ▪ “All of them are NFA [No Fixed Address] to begin with, so a lot of them…don’t really have a lot of community supports or connections or anything like that. Some of them have really acute health issues… [and] have been neglecting a lot of signs and symptoms for a really long time. So when they do come in, they need… follow up [care].” SMH-003 ▪ “I think everybody who’s vulnerable in the community, who are at risk for homelessness, who are at risk for being evicted from their homes, who do have medical conditions but there’s no way the doctors can communicate back with them… patients like this would definitely benefit from having [a] phone.” UHN-001
ED setting (familiar with serving underhoused patients)	▪ “In [our] ED, approximately 1 in 5 people are NFA [No Fixed Address]… they don’t have any way for [health or social] services to reach out to them… so they’re oftentimes lost to follow-up… I don’t think every single emergency department would really benefit from a program like this… but emergency departments where they have similar populations to ours, I feel like it makes a huge difference.” SMH-003 ▪ “We’re privy to knowing what people’s health challenges are, their housing challenges, and some of their social issues, so we're really the best fit to know how many appointments they really would need to connect with… often people are coming to the department just to seek shelter, so we’ve become somewhat of an assessment and referral center. So I think that we’re the best fit and we tend to be able to have psycho-social knowledge of what somebody’s going through, and what kind of emergencies they’re in.” SMH-001 ▪ “Emergency Medicine [is] where we are trying to close gaps that should be otherwise closed through other parts of the social infrastructure… [This program] is one of many stop gap measures that [we are using] to address poverty.” UHN-002
Staff approach	▪ “[The emergency department] is where social workers are always flagged for referrals into the community for seniors, people living alone, people isolated, people who are in the streets, people who have multiple comorbidities… So discharge planning [from the emergency department] was quite easy for me if I was I was involved with the patient, I had some understanding based on my psychosocial assessment that okay, “this patient has these issues and these are the challenges for discharge”, and I was able to connect the phone project.” UHN-001
Seamless program enrollment	▪ “It’s pretty streamlined, the process. We have had lots of support from our research coordinators to really help offload the emergency staff from being too involved… all you really do is identify the patient, and then trigger the process through our support workers who are generally the ones… to actually do all the steps to connect someone with the phone.” SMH-006 ▪ “Because of the step by step document that [describes how to activate a phone]… it’s very quick and efficient, and it doesn’t really take that much time. Like 5 to 10 min.” SMH-005 ▪ “I think it is very easy. Like when you [ask] somebody, "Hey, do you have connections to community? Do you have connections to family?" They're like, “No, I don’t have a phone”, and you’re like, “Oh, we have a phone program.” They’re very much interested. They very much are able to utilize it right there and then. So I find that it’s actually very easy to provide the service to clients.” SMH-002
Operational challenges	
Phone supply & activation in ED	▪ “The biggest challenge has been that there hasn’t always been a supply of phones… because it’s been something that someone has been doing off the side of their desk for two and a half years, and it’s not [an official, permanent program].” UHN-002 ▪ “What makes it difficult is the timing, that we’re in an [emergency department], people are leaving quickly, you know, we need the phone to be activated today.” SMH-002 ▪ “Things move really quickly in the emergency department, oftentimes patients come in and then they’re discharged within a few hours… oftentimes we’ll get our community support workers to activate the phone but it won’t be ready by the time the patient is discharged. So that means that they often have to come back the following day, and depending on their circumstances… that’s really difficult for them to do.” SMH-003
Eligibility criteria for enrollment	▪ “Right now, I feel like we're kind of picking and choosing who we give the phones to, which creates a whole population that are not getting the phones, which are people who are maybe experiencing more chronic homelessness, a bit more unorganized. And, probably we feel that they will not keep the phone or use the phone or, but that’s just kind of we’re judging them, which is not right at all. But, we’re trying to get the services to those people who are vocal in saying that, “I need the services.” And we’re trying to build relationships with those people.” SMH-002 ▪ “The pros of having criteria is it removes some of the ability for your own bias to interrupt who is getting phones and who's not. And I think bias is particularly strong when we’re thinking about folks experiencing poverty or homelessness, or marginalization.” UHN-002 ▪ “Sometimes people who are incredibly street entrenched, who are using substances on a regular basis… it’s going to be tricky for them to hold on to the same phone… That being said, I don’t think it’s not a good idea to give them a phone, because maybe that phone could be the key factor that allows them to connect to detox, or allows them to connect to supports and family.” SMH-003 ▪ “[A patients’] current socioeconomic position… could change in a few weeks and it would put them in a different situation. When we’ve been handing out phones it’s really low barrier, and the inclusion criteria is really anyone that care provider think needs a phone. And I think that’s ultimately best for now… [because] it’s hard to say… what position a patient [is] in in a small snapshot of their clinical setting. I think that can be problematic, and probably mostly inaccurate a lot of the time. So we can theorize who we think would benefit from it most, but you really may not know because your time with a patient is so finite in the ED.” SMH-006
Program communication	▪ “I think one challenge is that word definitely has gone around, that the emergency department is giving out phones. So, we’ll have people come in without registering to see a physician just to collect a phone… And we have to explain the purpose of the program, that we’re not able to do that.” SMH-003 ▪ “Somebody came in just to request a cell phone, but wouldn't indicate that until after the whole triage and two hours of sitting in the waiting room, which these days is not really something that we can accommodate.” UHN-002 ▪ “We have to really make it clear to the clients that this is a program and this is the reason I’m giving it to you. You know, you've got this follow up appointment, and I really want you to get to it. So then if people are coming in and being like, “I want a phone”, it’s like, “sorry they go to people who are have really complex health and a follow-up appointment.” SMH-001
Feasibility & sustainability	▪ “There’s a group of people that are very chronic and experiencing also chronic mental health, chronic addictions, and it’s harder for them to maintain the phone. It’s hard for them to keep the phone. So, sometimes I feel that I’m giving more phones to that person, or a few months and I’m giving them another phone, a few months, and I'm giving them another phone.” SMH-002 ▪ “People who lost phones, people who come back for replacements, what’s our policy going to be on that? If they’re not connected to a case manager, or just keeping in mind that it could increase visits for people or the ED… where people come who want their phone repaired or things like that.?” SMH-001

### Perceived outcomes

#### Building trust

Participants described the phone connect program as a tool to build trust with patients. By offering patients a phone, participants felt that they could demonstrate commitment to patients’ well-being and establish a trusting relationship. This foundation facilitated patients’ receptivity to the care offered, promoting uptake of the recommended healthcare services.

“It says a lot if someone says to you, ‘How am I going to organize my follow up?’ And you say ‘Well, I’m giving you a tool to do that.’…that builds a lot of trust… and I think that’s a facilitator of the entire rest of the engagement” University Health Network-002

Participants noted the program not only helped to build trust in their initial patient–provider interactions, but also facilitated long-term connection and support.

#### Facilitating patient independence

Upon building trust with patients, participants found that providing phones encouraged patients to take active roles in their care. While patients’ social situations can hinder their sense of independence, obtaining a phone can help them connect to services themselves and regain independence in their healthcare decisions.

“People are feeling neglected, forgotten about, excused. Phone-Connect provided them with… the opportunity to, on their own, choose if they wanted to continue on with their therapy, if they wanted to speak to someone.” St. Michael’s Hospital-004

Participants observed that phone provision also helped patients gain autonomy and independence in their personal lives, by accessing the internet or connecting with friends and family. Participants described the health benefits of social connection, noting the link between patients’ social connectivity and other positive outcomes, such as overcoming addictions.

#### Bridging care

Participants spoke extensively about their enhanced ability to follow up with patients, allowing them to effectively bridge care between the ED and other healthcare services. Since referral appointments are often finalized after discharge, providing phones helped to ensure patients successfully received those appointments and were connected to community services.

“Prior to Phone-Connect, if we were trying to connect somebody to a family doctor or to a caseworker, oftentimes that connection would never happen because they leave the emergency department… [and] they’re moving from shelter to shelter, so there’s absolutely no way for people to get a hold of them.” St. Michael’s Hospital-003

Due to improved patient follow-up, participants observed increased access to services, such as shelter, medical appointments, legal services, and case workers, with many describing specific examples of patients who benefited from the program. Participants noted the potential system impacts of patients’ increased access to services, including reducing unnecessary ED visits, since patients “don’t have to utilize the [ED] for their primary health care” (St. Michael’s Hospital-002), and potentially reducing hospital admissions.

#### Enabling equitable care for marginalized populations

Participants emphasized that providing phones is an essential first step in adequately serving patients experiencing marginalization. They stated the program enabled effective, equitable care for these patients by meeting some of their unique needs and addressing social determinants of health:

“It’s social supports, medical services, but all in all, it’s about… the social determinants of health. If people don’t have access to a GP, or a case worker or wound care, it ultimately is going to… impact their physical health down the road.” St. Michael’s Hospital-003

Participants conveyed the integral nature of phones for effectively treating patients, with one participant noting, “without the phone, it is very difficult to do this job” (St. Michael’s Hospital -002). Some noted that phone provision enables effective discharge and safety planning for patients experiencing marginalization, as phone possession is fundamental to these plans.

#### Mitigating moral distress among healthcare workers

By being able to provide effective, equitable care, participants expressed the program’s positive impacts on their own mental well-being and job satisfaction. Participants felt that the program supported their delivery of care by providing a tool to treat patients, alleviating their discouragement at being chronically unable to adequately serve this population:

“[The program] was very beneficial in the sense that it provided me with tools... Because not only can [homelessness and marginalization] be discouraging for… the client, it can be discouraging for your staff as well, because at some points you start to feel like you’re just pushing a rock uphill that's really slowly crushing you.” St. Michael’s Hospital-004

Participants noted that giving healthcare workers concrete tools helped mitigate the broader moral distress often experienced in emergency medicine.

### Contextual enablers

Participants highlighted contextual factors that were key to program success (Table 3). Importantly, the program predominantly served underhoused patients experiencing complex, long-standing health and social issues, requiring specific follow-up care; the program was delivered in EDs well-versed in serving these populations. The healthcare workers in these EDs are familiar with the unique needs of this patient population and treat patients with “an open, non-judgmental, anti-oppressive framework” (St. Michael’s Hospital -001), which they felt was essential to building trust and connection. Seamless program enrollment was also felt to be critical for maintaining ED workflow while effectively serving patients.

### Operational challenges

Participants discussed challenges and suggestions for program operations (Table 3). Program administration had not been fully formalized, so phone supply was inconsistent and eligibility criteria for enrollment were unclear, leaving staff uncertain as to who should receive phones and how many replacement phones could be given if lost. Participants suggested that more specific criteria and communication could clarify these parameters and mitigate potential bias in administering the program. However, they noted that determining criteria is difficult and nuanced, as it is unclear who should be excluded; while some patients’ precarious situations may increase their likelihood of losing phones, they may also benefit most from targeted follow-up care. Participants conveyed wanting to keep a “low barrier” for enrollment but recognized the need to prioritize patients due to resource constraints. They also noted that patients seeking phones might contribute to unnecessary ED visits and long ED wait times. Given these issues, as well as questions about ongoing technical support for the phones, some participants expressed feasibility and sustainability concerns; however, they emphasized that the operational challenges encountered were minimal compared to the program’s benefits.

## Discussion

### Interpretation

Our examination of healthcare workers’ perspectives suggests that providing mobile phones to patients experiencing marginalization in the ED may lead to important patient and system outcomes, including fostering trust with patients, facilitating patient engagement in healthcare, increasing access to services across care sectors, enabling equitable care for marginalized patients, and mitigating moral distress among healthcare workers. Our study highlights the interrelatedness of these outcomes and the influence of contextual and operational factors on program implementation and outcomes. Notably, simply providing phones may be insufficient; delivering the program in settings familiar with the care of marginalized patient populations, and establishing trusting relationships with patients using non-judgmental, anti-oppressive approaches, may facilitate its success. Additionally, ensuring seamless program enrollment within the ED through adequate phone supply, defined eligibility criteria, and clear program communication may support sustainability. Overall, participants expressed that phone provision is invaluable for patients facing barriers to accessing healthcare and social services, and for healthcare workers who lack resources to adequately serve marginalized populations.

### Comparison to previous studies

This is one of the first peer-reviewed evaluations of a prescription mobile phone program for patients in the ED experiencing marginalization. Underhoused patients often face significant challenges with discharge and follow-up as discharge plans rarely account for risk factors associated with homelessness [12, 13]. Participants in this study reiterated that many discharge plans are ineffective for patients without phones, and that providing phones enabled successful follow-up and increased continuity of care across sectors, reducing patient reliance on the ED and potentially decreasing hospital admissions. Phone provision reportedly facilitated access to social services such as shelter, case workers, and legal aid. It also seemed to enhance patients’ social connectivity, which has considerable health benefits [28, 29], and may be invaluable for people experiencing homelessness who are susceptible to social isolation [30].

For patients experiencing homelessness, lack of trust is a common barrier to accessing care, whereas positive relationships with healthcare workers can facilitate healthcare utilization [3, 33]. Patients experiencing marginalization often feel unwelcome in healthcare environments, deterring them from engaging in healthcare [6, 36]; building trust using non-judgmental, anti-oppressive approaches is imperative [33, 37]. In this study, participants felt that this relational approach was foundational to the program, as phones were given in the context of empathetic interactions which promoted trust and encouraged patients’ engagement in the healthcare referrals. Such active engagement can improve treatment adherence, health outcomes, and satisfaction with care [38]. Additionally, building trust and reducing digital health inequities were rewarding for healthcare workers. Whereas experiencing patient mistrust and feeling constrained in providing adequate care can lead to moral distress [39, 40], having concrete tools to serve patients may facilitate fulfillment among staff.

Despite the positive perceived outcomes of the program, participants highlighted operational challenges that require attention. Notably, while they emphasized the importance of minimizing barriers to access, they recognized the need to put parameters around phone provision to ensure program feasibility and sustainability. Moreover, the absence of enrollment criteria could put healthcare workers in the undesirable position of determining who receives a phone, and potentially introduce bias into program administration. Implicit biases can influence clinical decision-making and outcomes, particularly in fast-paced, high-pressure settings [41] such as EDs. In response to these concerns, the eligibility criteria were subsequently narrowed to focus on patients newly connected to case managers, community health programs, or the hospitals’ addictions clinics, as these patients are the “highest risk repeat users” of the ED and have assigned follow-up workers who can aid their use of the program. This change in criteria aimed to facilitate integration of the program with minimal burden on ED staff [42], while prioritizing the highest need patients, recognizing that those at greatest risk of losing a phone are often those most in need of one.

### Strengths and limitations

This was among the first studies to evaluate a prescription phone program. Our sample was small; however, we generated rich data and our rigorous reflexive analysis produced insights that may be transferrable in other contexts with similar patient populations. Our sample was predominantly female, reflective of the demographic profile of the program staff. Finally, our study focused on healthcare workers’ perspectives, which was an important first step in understanding whether and how the program works, and which factors may influence future implementation; patient perspectives and outcome measures will be examined subsequently to further elucidate program benefits and challenges.

### Health system implications

Our findings have clinical implications for addressing health inequities and practical implications for future implementation efforts. Notably, providing phones should be in the context of a *program*, inclusive of relational care and a biopsychosocial understanding of health. Embedding this program in a setting well-versed in serving people experiencing homelessness and marginalization may facilitate its success [7]. Additionally, maintaining efficient ED workflow is important for sustainability; therefore, seamless program enrollment and clear communication about program purpose and eligibility criteria are needed. Future implementation efforts should focus on clear criteria that facilitate efficient and sustainable program enrollment while maintaining low barriers to phone access. Attention to possible bias in program administration may be necessary, particularly if implemented in contexts unfamiliar with treating patients experiencing marginalization. Overall, a non-judgmental approach to providing phones and replacing them if lost is encouraged.

### Research implications

The benefits of addressing social determinants of health are well established [33–35], and the need to consider digital determinants of health is increasingly recognized [16, 21]. Our findings add to this knowledge base by demonstrating the positive perceived effects of providing phones to patients experiencing marginalizing conditions. Continued delivery and evaluation of prescription phone programs will build further knowledge on their potential role in addressing health inequities. Future longitudinal research is needed to examine the social, contextual, and operational factors that facilitate patient adherence and outcomes. Further research is also needed to explore patient perspectives and outcomes to comprehensively understand program impact.

## Conclusion

We qualitatively evaluated an ED-based prescription mobile phone program for patients experiencing homelessness and marginalization in two urban hospitals from the perspective of healthcare workers. Our study highlighted positive perceived effects of mobile phone provision in addressing digital and social health inequities, and illuminated critical contextual factors to inform new iterations of the program. Ongoing research on patient perspectives and outcomes will further our understanding of the role of mobile phone provision in facilitating access to care and combating health inequities among marginalized populations.
